# Potential correlation between dental caries and intracranial aneurysm: an innovative prognostic marker for intracranial aneurysm development

**DOI:** 10.3389/fneur.2025.1561207

**Published:** 2025-05-23

**Authors:** Jia Sun, Siming Gui, Dachao Wei, Jia Jiang, Jun Lin, Wentao Gong, Huijian Ge, Youxiang Li

**Affiliations:** ^1^Tianjin Key Laboratory of Oral and Maxillofacial Function Reconstruction, Department of the First Clinical Division, Tianjin Stomatological Hospital, School of Medicine, Nankai University, Tianjin, China; ^2^Department of Neurointerventional Surgery, Beijing Neurosurgical Institute, Capital Medical University, Beijing, China; ^3^Beijing Engineering Research Center for Interventional Neuroradiology, Beijing, China; ^4^Department of Interventional Neuroradiology, The First Affiliated Hospital of Zhengzhou University, Zhengzhou, China; ^5^China National Clinical Research Centre for Neurological Diseases, Department of Neurosurgery, Beijing Tiantan Hospital, Capital Medical University, Beijing, China

**Keywords:** intracranial aneurysm, subarachnoid hemorrhage, dental caries, oral infection, root canal treatment

## Abstract

**Background:**

The prevalence of intracranial aneurysm (IA) in the population is approximately 3–7%, with a rupture mortality rate as high as 40%. Identification of risk factors for IA occurrence and provision of targeted preventive and therapeutic measures are crucial for clinical diagnosis of IA. Dental caries is a common oral disease that affects the global population. In this study, we aimed to explore the potential connection between dental caries and the incidence of IA.

**Methods:**

We conducted a single-center retrospective 1:1 matched case-control study to assess the correlation between dental caries and the occurrence of IA among 230 participants. Participants were categorized into IA and non-IA groups. All participants underwent cerebral digital subtraction angiography or magnetic resonance angiography, as well as oral assessment. Using binary logistic regression analyses, we examined whether presence of dental caries was correlated with the occurrence of IA.

**Results:**

Compared with the non-IA group, the IA group exhibited a greater prevalence of dental caries (90.44% vs. 56.52%) and a greater prevalence of history of root canal treatment (73.91% vs. 48.70%). Multiple logistic regression analysis revealed a significance between the presence of dental caries (OR: 4.14, 95% CI: 1.35–12.66) and IA occurrence. Also, the history of root canal treatment (OR: 2.03, 95% CI: 1.09–3.79) were significantly associated with IA occurrence (all *p* < 0.05).

**Conclusion:**

Dental caries was significantly associated with the incidence of IA. Cariogenic bacteria may enter the systemic circulation through pulp, potentially leading to pathological changes in normal cerebral blood vessels, such as the development of IA.

## Introduction

Intracranial aneurysm (IA) is prevalent in approximately 3–7% of the general population; it consists of pathological dilations of cerebral arteries that are often characterized by abnormal sac-like protrusions at proximal bifurcations of these arteries (1). The incidence of subarachnoid hemorrhage (SAH) caused by rupture of IA was reported to be 10 to 11 cases per 100,000 individuals per year in multiple countries (2). The mortality rate associated with IA rupture and subsequent SAH can be as high as 35%, with the majority of survivors experiencing severe neurological deficits (3, 4). Endovascular intervention or neurosurgery is the standard approach for treating unruptured IA, aimed at reducing the risk of SAH resulting from IA rupture. However, a significant proportion of unruptured IAs are asymptomatic and undetected by patients. Therefore, accurate diagnosis of IA before rupture is crucial, and a clear understanding of the pathophysiological mechanisms underlying IA formation will greatly assist in clinical diagnosis and treatment.

Chronic inflammation mediated by inflammatory cells and subsequent remodeling of cerebral arteries have emerged as critical factors in the formation of IA and the subsequent degeneration of IA walls, ultimately leading to rupture (5–8). Previous research has confirmed the presence of bacterial DNA within IA walls, along with the expression of Toll-like receptors (TLRs) that recognize bacterial components and trigger an immune response (9). These findings strongly link pathogens from oral infections to the inflammation-mediated remodeling of arterial and IA walls, resulting in IA development and subsequent rupture, leading to SAH. Furthermore, real-time quantitative polymerase chain reaction analyses have identified bacterial DNA from various oral pathogens (9), suggesting potential connections between different oral diseases and the occurrence of IA.

*Streptococcus mutans* is a well-established dental pathogen associated with caries, a prevalent dental affliction that results in tooth decay and, if left untreated, inflammation and necrosis of the neural and vascular structures of the tooth pulp (10). These complications can culminate in root canal treatment or extraction of the affected tooth, often leading to tooth loss. Certain strains of *S. mutans*, such as *Cnm* and *Cbm*, express proteins capable of binding to collagens and are linked to a more aggressive form of caries (11). Notably, *S. mutans* expressing collagen-binding protein (CBP) showed a higher prevalence in oral samples from patients with cerebral hemorrhage (12, 13) and other forms of intracranial hemorrhage, including SAH (14). In animal models, *S. mutans* CBP was identified as a predisposing factor for intracranial hemorrhage by exerting a direct effect on the cerebral artery wall, with being CBP detectable in the cerebral hemorrhage tissues after oral administration (12). Therefore, this pathogen, which plays a crucial role in caries development, may also be involved in the formation of IA.

Given previous research indicating the presence of cariogenic pathogens in the tissue of IA walls (9), our objective was to investigate the potential correlation between dental caries and the risk of IA development.

## Materials and methods

### Study population

From September 2018 to September 2021, we recruited patients admitted to our institution for the diagnosis of unruptured IA by digital subtraction angiography (DSA). During the same period, we also recruited healthy volunteers without IA. A total of 561 participants signed written informed consent forms to participate in the study and were divided into an IA group and a non-IA group. Patient privacy was strictly protected, and the study protocol was approved by our institutional ethics committees (IRB of Beijing Tiantan Hospital and IRB of Tianjin Stomatological Hospital).

The inclusion criteria were as follows: (1) individuals aged 18–80 years; (2) individuals with unruptured IA diagnosed by DSA and healthy volunteers without IA detected by magnetic resonance angiography (MRA) at our medical center during routine physical examination who wished to participate in the study; (3) individuals who underwent oral assessment.

The exclusion criteria were as follows: (1) individuals found to have a combination of severe gingivitis and/or periodontitis by oral assessment; (2) individuals who had received antibiotic treatment in the past 3 months; (3) individuals who had undergone dental caries treatment in the past 3 months; (4) female subjects who were pregnant or planning to become pregnant during the study period; (5) individuals with ruptured IA diagnosed by DSA with or without a history of surgical intervention; (6) individuals with a modified Rankin Scale score ≥3, indicating an inability to live independently (0 = completely asymptomatic, 1 = symptomatic but without significant functional impairment, able to perform all daily activities and work, 2 = mild disability, unable to perform all activities but can manage personal affairs without assistance, 3 = moderate disability, requiring some help but able to walk independently, 4 = moderately severe disability, unable to walk independently, requiring assistance in daily life, 5 = severe disability, bedridden, incontinent, completely dependent on others for daily activities); (7) individuals with intracranial masses (tumors, meningiomas, brain abscesses, other infections) or undergoing radiation and chemotherapy for head or neck cancer or sarcoma; (8) individuals with comorbidities that may result in survival for <12 months; (9) individuals who had undergone extracranial (carotid or vertebral artery) or intracranial stent placement, angioplasty, or intramural dissection surgery in the past 30 days; (10) individuals with other known severe complications such as heart disease (atrial fibrillation/pacemaker, recent myocardial infarction, symptomatic congestive heart failure), lung disease, uncontrolled diabetes, progressive neurological disorders, vasculitis; (11) individuals who had received immunosuppressive agents, including corticosteroids in the past 3 months; (12) individuals with aneurysms in non-intracranial arteries, Ehlers–Danlos syndrome, or Marfan syndrome.

### Assessment of dental caries

Participants who agreed to undergo a dental examination were subjected to a comprehensive assessment. The dental examination was conducted free of charge for all recruited individuals through a standardized approach in a dental unit using a standard dental light, compressed air, a mouth mirror, and digital panoramic radiography. A thorough cleaning of the teeth was performed before the examination to remove plaque, calculus, and debris that might obscure the dentist’s view or interfere with diagnostic procedures. Participants were lying on a dental chair, under a light. The dentist uses a dental mirror and probe to visually inspect the teeth for signs of decay, cracks, or other abnormalities. Dental caries was diagnosed using World Health Organization (WHO) diagnostic criteria. The assessment involved the evaluation of cavitations, defined as teeth exhibiting enamel surface discontinuity due to a loss of the tooth structure. Each tooth was assigned a numerical code to indicate its status: sound, decayed or filled, missing, restored with crown, decayed root fragment, sound root fragment, or implant. Teeth with a status code of 2 (decayed or filled) or 4 (restored with crown) were categorized as dental surface caries and/or dental root caries. The presence of root caries was recorded if a discrete, well-defined, and discolored cavitation was observed on the root surface and if the dental explorer easily entered the cavitation. Root caries was scored if at least half of the lesion or restoration extended apically beyond the cementoenamel junction. It should be noted that fractures, erosions, and abrasions were considered separate entities and were not documented as caries. Participants with non-cavitated lesions underwent further investigations to detect pit, fissure, and smooth surface lesions as well as root and coronal caries. A graduated manual periodontal probe was used to measure four sites (buccal, mesiolingual, lingual, and distolingual) of the distance between the pocket and cementoenamel junction per tooth. Periodontitis was evaluated by using the clinical attachment loss (CAL) was scored at four sites per tooth and averaged after rounded to the nearest millimeter for each participant. Attachment levels were analyzed as continuous variables, and mean CAL >4 mm was considered as severe periodontitis. Gingivitis severity was assessed by using the gingival index (GI) system based on the various tendencies of gingival bleeding after probing (0 = normal gingiva; 1 = mild inflammation: slight change in color, slight oedema. No bleeding on probing; 2 = moderate inflammation: redness, oedema and glazing. Bleeding on probing; 3 = severe inflammation: marked redness and oedema. Ulceration; tendency to spontaneous bleeding). A high index (>1.0) was defined as severe gingivitis.

### Clinical data collection

We collected clinical baseline data and imaging information from cerebral DSA and MRA for the study participants diagnosed with or without IA at Beijing Tiantan Hospital, Capital Medical University, through the electronic medical record system. Smokers were defined as individuals currently smoking or those with a history of smoking. Participants with untreated diabetes, those using insulin or oral hypoglycemic agents, or those diagnosed with diabetes by a physician were considered diabetic. Individuals with untreated hypertension, those using antihypertensive medications, or those diagnosed with hypertension by a physician were classified as hypertensive. Participants with a history of hyperlipidemia or diagnosed with hyperlipidemia by a physician were considered to have hyperlipidemia. Individuals with a history of myocardial infarction, angina, percutaneous coronary intervention, or coronary artery bypass graft surgery were classified as having heart disease. Imaging parameters for IA, including maximum diameter, number, morphology, and location, were manually measured and recorded by two experienced neurointerventionalists based on previously published measurement methods. Morphology of IA was categorized as saccular or non-saccular based on 3D-DSA reconstructed images. Location of IA was classified into bilateral internal carotid arteries (cavernous, clinoid, ophthalmic, and posterior communicating segments), bilateral posterior communicating arteries, bilateral anterior cerebral arteries, anterior communicating artery, bilateral middle cerebral arteries, bilateral posterior cerebral arteries, basilar artery (including bilateral superior cerebellar arteries and anterior inferior cerebellar arteries), and bilateral vertebral arteries (intracranial segment and bilateral posterior inferior cerebellar arteries). Morphological parameters for IA were defined as follows: maximum diameter, measured as the largest diameter of the IA on 2D- or 3D-DSA. All IA images were independently interpreted by two neurointerventionalists, ensuring that they were blinded to each other’s interpretations and the patients’ clinical information. Discrepancies in interpretation were resolved by a third adjudicating neurointerventionalist.

### Statistical analysis

We conducted propensity score matching (PSM) to perform 1:1 matching on IA and one non-IA group with the psmpy package (version 0.3.13) used for python (version 3.11.4), categorized by age, gender, and educational background in order to create comparable baseline characteristics of participants in each group. Data are presented as median [IQR] or frequency and percentage. The normality of the variables was assessed using the Shapiro–Wilk test. An independent-samples *t*-test, Mann–Whitney *U* test, and the chi-square test were used to analyze the differences in continuous and categorical variables between the IA and non-IA groups, respectively. Univariate and multivariate logistic regression analyses were performed to determine the independent contribution of dental caries to the incidence of IA. Variables with variance inflation factors >10 in the collinearity test were excluded from the logistic regression analysis. Odds ratios (ORs) and 95% confidence intervals (CIs) were calculated for all parameters. A two-tailed *p*-value of <0.05 was considered significant. All statistical analyses were conducted using SPSS 22.0 software (SPSS Inc., Chicago, IL, United States).

## Results

**Figure 1:**
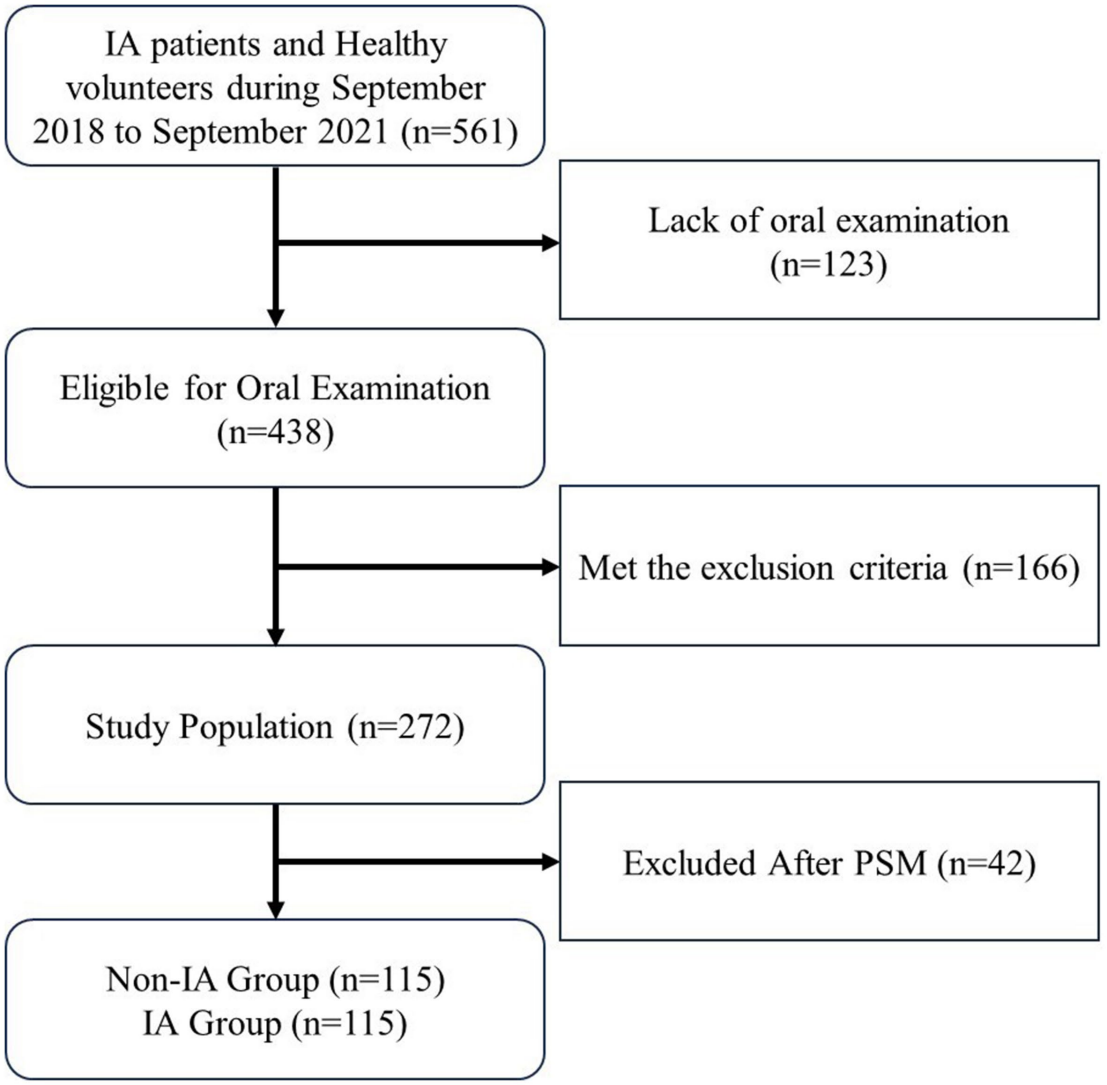
Flowchart of sample selection. Study population selection (*n* = 230). IA, intracranial aneurysm; PSM, propensity score matching.

Figure 1 illustrates the process of screening participants for this study. The study eventually enrolled a total of 230 participants, with 115 participants in each group, comprising 149 women (64.78%) and 81 men (35.22%). The age of the participants ranged from 18 to 79 years, with a mean age of 51 [43, 58] years. The baseline characteristics and subgroup analysis results for the IA and non-IA groups are detailed in Table 1. There were no significant differences in baseline (age, gender, educational background, personal income, smoking, alcohol consumption, and BMI) and clinical characteristics (hypertension, diabetes mellitus, dyslipidemia, carotid artery stenosis and dissection, history of ischemic stroke, history of cerebral hemorrhage, history of subarachnoid hemorrhage and family history of IA) between the two groups of patients. However, significant differences were observed in variables related to dental caries between the non-IA and IA groups. These variables included dental caries (56.52% vs. 90.44%), number of dental caries (1 [0, 2] vs. 2 [1, 2]), coronal caries (55.65% vs. 87.83%), number of coronal caries (1 [0, 2] vs. 1 [1, 2]), root caries (4.35% vs. 16.52%), number of root caries (0 [0, 0] vs. 0 [0, 0]), crown replacement (21.74% vs. 46.96%), number of replaced crown (0 [0, 0] vs. 0 [0, 2]), root canal treatment (48.70% vs. 73.91%), number of teeth for root canal treatment (0 [0, 3] vs. 2 [0, 4]), and decayed, missing, and filled teeth (DMFT) (2 [0, 5], 3 [1, 5]) (all *p* ≤ 0.05).

**Table 1:** Baseline characteristics of the participants.

Variables	Total (*n* = 230)	Non-aneurysm group (*n* = 115)	Aneurysm group (*n* = 115)	*p*-value^a^
Age	51 [43, 58]	50 [41, 57]	52 [46, 58]	0.052
Female	149 (64.78%)	75 (65.21%)	74 (64.34%)	0.890
High school educational background	78 (33.91%)	42 (36.52%)	36 (31.30%)	0.403
Personal income <1,000 USD/month	150 (65.21%)	69 (60.00%)	81 (70.43%)	0.064
Hypertension	75 (32.61%)	34 (29.57%)	41 (35.65%)	0.325
Diabetes mellitus	26 (11.30%)	15 (13.04%)	11 (9.57%)	0.405
Dyslipidemia	17 (7.39%)	9 (7.83%)	8 (6.96%)	0.801
Carotid artery stenosis and dissection	24 (10.43%)	11 (9.56%)	13 (11.30%)	0.415
History of ischemic stroke	12 (4.6%)	7 (6.09%)	5 (4.35%)	0.553
History of cerebral hemorrhage	2 (0.87%)	1 (0.87%)	1 (0.87%)	1.000
History of subarachnoid hemorrhage	26 (11.30%)	13 (11.30%)	13 (11.30%)	1.000
Smoke	41 (17.83%)	18 (15.65%)	23 (20.00%)	0.389
Alcohol consumption	31 (13.48%)	20 (17.39%)	11 (9.57%)	0.082
Family history of intracranial aneurysm	22 (9.57%)	9 (7.83%)	13 (11.30%)	0.370
Body mass index	24.56 [22.60, 27.04]	24.45 [21.55, 27.36]	24.67 [23.23, 26.95]	0.327
Teeth number	30 [28, 31]	30 [28, 31]	29 [27, 31]	0.486
Number of missing teeth	1 [0, 2]	0 [0, 2]	1 [0, 2]	0.515
Implant teeth	19 (8.26%)	8 (6.96%)	11 (9.57%)	0.472
Number of implant teeth	0 [0, 0]	0 [0, 0]	0 [0, 0]	0.435
Dental caries	169 (73.48%)	65 (56.52%)	104 (90.44%)	**<0.001**
Number of caries	1 [0, 2]	1 [0, 2]	2 [1, 2]	**<0.001**
Coronal caries	165 (71.74%)	64 (55.65%)	101 (87.83%)	**<0.001**
Number of coronal caries	1 [0, 2]	1 [0, 2]	1 [1, 2]	**<0.001**
Root caries	24 (10.44%)	5 (4.35%)	19 (16.52%)	**0.003**
Number of root caries	0 [0, 0]	0 [0, 0]	0 [0, 0]	**0.002**
Caries filled treatment	39 (16.96%)	14 (12.17%)	25 (21.74%)	0.053
Number of filled caries	0 [0, 0]	0 [0, 0]	0 [0, 0]	0.063
Crown replacement	79 (34.35%)	25 (21.74%)	54 (46.96%)	**<0.001**
Number of replaced crowns	0 [0, 1]	0 [0, 0]	0 [0, 2]	**<0.001**
Root canal treatment	141 (61.30%)	56 (48.70%)	85 (73.91%)	**<0.001**
Number of teeth for root canal treatment	1 [0, 4]	0 [0, 3]	2 [0, 4]	**0.001**
Dental extraction	112 (48.70%)	57 (49.57%)	55 (47.83%)	0.792
Number of extracted teeth	0 [0, 2]	0 [0, 2]	0 [0, 2]	0.995
Decayed, missing, and filled teeth	2 [1, 5]	2 [0, 5]	3 [1, 5]	**0.007**
Gingival index	0.58 [0.26, 0.98]	0.49 [0.26, 0.76]	0.73 [0.26, 1.03]	0.138
Clinical attachment loss	3.14 [2.47, 3.46]	3.14 [2.42, 3.36]	3.14 [2.67, 3.75]	0.442

a *p*-values were calculated for categorical covariates using the chi-square test, whereas *p*-values were calculated using the Student’s *t*-test and Mann–Whitney *U* test for continuous variables. Median [IQR] for the continuous variables. *N* (percentage) for the categorical variables, significance level *p* < 0.05.

The results for the aneurysm characterization in the IA group are shown in Table 2. For the aneurysm locations, the internal carotid artery is the most common location (60.0%) for IA occurrence. In terms of aneurysm size, most participants (88.7%) had small (<7 mm) and medium-sized (7–15 mm) aneurysms. The participants’ aneurysm morphology was predominantly saccular (72.2%), and the majority of participants had single IA (80.9%).

**Table 2:** Aneurysm characteristics in the IA group.

	Number	Proportions
Size (diameter) of intracranial aneurysm^a^		
<7 mm	47	40.9%
7–15 mm	55	47.8%
>15 mm	13	11.3%
Total	115	100.0%
Location of intracranial aneurysm^a^		
Anterior cerebral artery	4	3.5%
Middle cerebral artery	7	6.1%
Anterior communicating artery	7	6.1%
Posterior communicating artery	4	3.5%
Internal carotid artery	69	60.0%
Posterior cerebral artery	2	1.6%
Basilar artery	8	7.0%
Vertebral artery	14	12.2%
Total	115	100.0%
Number of intracranial aneurysms		
Single	93	80.9%
Multiple	22	19.1%
Total	115	100.0%
Shape of intracranial aneurysm^a^		
Saccular	83	72.2%
Fusiform	32	27.8%
Total	115	100.0%

a If the patient had multiple intracranial aneurysms, the size, location, and shape of the largest aneurysm were recorded.

Through co-linear analysis, we excluded variables with a variance inflation factor (VIF) greater than 10 before binary logistic regression analyses, which included coronal caries, root caries, DMFT, the number of teeth, the number of implant teeth, the number of caries (including coronal caries and root caries), the number of extracted teeth, and the number of treated teeth (including filling treatment, root canal treatment, and crown replacement). Several factors significantly associated with the occurrence of IA were identified in the univariate logistic regression analyses (Table 3). These factors included advanced age (OR: 1.04, 95% CI: 1.01–1.06, *p* = 0.006), dental caries (OR: 7.27, 95% CI: 3.53–14.98, *p* < 0.001), history of crown replacement (OR: 3.19, 95% CI: 1.79–5.66, *p* < 0.001), and root canal treatment (OR: 2.99, 95% CI: 1.72–5.19, *p* < 0.001).

**Table 3:** Univariate binary logistic regression analyses of clinical characteristics and dental parameters for intracranial aneurysm formation.

Variable	*p* ^a^	OR	95% CI	
			Lower	Upper
Age	**0.006**	1.04	1.01	1.06
Female	0.890	1.04	0.60	1.78
High school educational background	0.404	0.79	0.46	1.37
Personal income <1,000 USD/month	0.098	0.63	0.36	1.08
Diabetes mellitus	0.406	0.71	0.31	1.61
Hypertension	0.325	1.32	0.76	2.30
Dyslipidemia	0.801	0.88	0.33	2.37
Carotid artery stenosis and dissection	0.667	1.20	0.51	2.81
History of ischemic stroke	0.555	0.70	0.22	2.28
History of cerebral hemorrhage	1.000	1.00	0.06	16.18
History of subarachnoid hemorrhage	1.000	1.00	0.44	2.26
Smoke	0.390	1.35	0.68	2.66
Alcohol consumption	0.086	0.50	0.23	1.10
Family history of intracranial aneurysm	0.372	1.50	0.62	3.66
Body mass index	0.313	1.03	0.97	1.10
Number of missing teeth	0.714	0.99	0.94	1.04
Implant teeth	0.474	1.41	0.55	3.66
Dental caries	**<0.001**	7.27	3.53	14.98
Dental extraction	0.792	0.93	0.56	1.56
Caries filled treatment	0.056	2.00	0.98	4.09
Crown replacement	**<0.001**	3.19	1.79	5.66
Root canal treatment	**<0.001**	2.99	1.72	5.19
Crown caries remnant	0.209	4.11	0.45	37.29
Gingival index	0.120	1.66	0.88	3.13
Clinical attachment loss	0.215	1.23	0.88	1.72

a Univariate binary logistic regression, significance level *p* < 0.05. OR, odds ratio; CI, confidence interval.

To further clarify the correlation between dental caries or related factors and the occurrence of IA, we adjusted for significant variables (*p* ≤ 0.05) identified in the univariate logistic analyses and incorporated them into a multivariate logistic regression analysis (Table 4). The multivariate logistic regression analysis demonstrated that dental caries (OR: 4.14, 95% CI: 1.35–12.66) and root canal treatment (OR: 2.03, 95% CI: 1.09–3.79) continued to play a role in the formation and development of IA (all *p* ≤ 0.05).

**Table 4:** Multivariate binary logistic regression of dental caries parameters for intracranial aneurysm formation.

Variable	*p* ^a^	Adjusted OR	95% CI	
			Lower	Upper
Age	0.357	1.02	0.98	1.05
Dental caries	**0.013**	4.14	1.35	12.66
Crown replacement	0.493	1.40	0.54	3.62
Root canal treatment	**0.027**	2.03	1.09	3.79

a Multivariate binary logistic regression, significance level *p* < 0.05. OR, odds ratio; CI, confidence interval.

## Discussion

The present study revealed an independent correlation between dental caries and IA occurrence. Dental caries, a prevalent chronic condition characterized by microbiological biofilm formation, is an important oral health concern. During the initial stages, caries is amenable to reversal; however, it can advance, leading to substantial tooth damage and potential loss. Dental caries represents a primary cause of oral discomfort and eventual tooth loss and typically exhibits gradual progression (15, 16). It is a widespread ailment that afflicts adults worldwide, representing a significant public health concern owing to its well-established links with cardiovascular disease (17), cerebrovascular disease (12, 14, 18), and diabetes mellitus (19, 20). Numerous recognized risk factors contribute to dental caries. Physical determinants include insufficient salivary flow and elevated levels of cariogenic bacteria, while lifestyle factors encompass suboptimal oral hygiene practices and socioeconomic status disparities (15, 16). Our results highlight an association between dental caries and IA occurrence, suggesting that mitigating risk factors for dental caries may concurrently attenuate susceptibility to IA.

Finnish researchers conducted an investigation into the potential correlation between dental caries and unruptured IA (21). Their findings indicated that dental caries had no significant association with unruptured IAs. Intriguingly, their study revealed an inverse relationship between caries and IA occurrence. This inverse relationship may be attributed to the fact that symptomatic caries often prompts dental care-seeking behaviors, consequently leading to the management of other oral conditions, such as periodontitis. It is noteworthy that dental caries is the most prevalent dental ailment globally, affecting nearly 3.5 billion individuals worldwide (22). While the incidence of caries was found to be relatively higher in high socioeconomic status populations (22), a study conducted in that demographic population did not identify caries as a significant factor associated with an elevated risk of unruptured IA (21). The authors posited several plausible rationales for the absence of an association. First, caries is inherently a localized infectious ailment that primarily affects the tooth structure, with systemic implications typically arising only in cases where caries penetrates the tooth pulp. Second, caries is frequently addressed through restorative interventions like fillings, often before it progresses to severe conditions such as pulp inflammation. Third, unlike periodontal diseases affecting the gingival tissues, caries does not tend to predispose individuals to bacteremia.

However, in contrast to the observations of the Finnish researchers (21), the present study established an association between the incidence of IA and dental caries. We propose several plausible explanations for this discrepancy. In China, as a developing nation, there exists a lower level of awareness regarding dental health protection, potentially resulting in instances where individuals seek medical attention for dental caries only when it has reached an advanced stage. This, in turn, may elevate the risk of oral infections disseminating to the cerebral vasculature via the bloodstream. In our study, over 65% of participants had a monthly income of less than $1,000, and only 1/3 of our participants had a high-school education background. Additionally, a higher percentage of patients in the IA group underwent root canal treatment, which also confirms the point above. On the contrary, unruptured IAs are frequently identified during routine physical examinations because they often manifest without specific symptoms. In their demographic population, concurrent oral examinations conducted during physical check-ups can readily detect dental caries. Moreover, variations in ethnicity, geography, and dietary practices may introduce interactions between oral flora and cariogenic bacteria, potentially modulating the impact of cariogenic bacteria on IA. However, it is important to note that this hypothesis requires substantiation through subsequent research endeavors.

Earlier investigations established an association between the presence of *S. mutans* and a reduced time frame for caries development. Furthermore, *Cnm*-positive *S. mutans* has been recognized for its role in promoting intracranial hemorrhage by directly impeding platelet aggregation, as documented in several reports (12, 13, 18, 23). Nonetheless, the connection between antiplatelet effects and aSAH remains a topic of uncertainty. It is noteworthy that a previous study concluded that use of antiplatelet agents did not elevate the risk of aSAH (24). In light of these considerations, the present study refrained from investigating a distinct subgroup of patients with ruptured IA. Instead, the primary focus was to investigate the potential relevance of dental caries to the initiation and progression of IA.

In our cohort of patients with IA, we observed that those with a history of root canal treatment were more likely to develop IA (adjusted OR: 2.03, 95% CI: 1.09–3.79, *p* = 0.027). This association may reflect a chronic, low-grade inflammatory state mediated by oral pathogens. The progression of untreated dental caries can lead to recurrent bacteremia, the destruction of dental hard tissues results in pulpal infection, allowing bacteria and their metabolites (e.g., lipopolysaccharides, collagen-binding proteins) to enter the systemic circulation, promoting systemic inflammation (1). Additionally, DNA of cariogenic microorganisms has been identified in specimens of IA walls (9), suggesting that bacteria and their metabolites eventually migrate to the cerebral vasculature, forming a localized inflammatory response. The inflammatory process induced by oral pathogens is thought to affect the arterial wall by triggering immune responses through the activation of TLRs, which are known to recognize bacterial components (1, 9, 25). This inflammation could exacerbate endothelial dysfunction in cerebral arteries, leading to vascular remodeling and the formation of IA (5, 7). However, it is important to note that while the presence of oral pathogens in the cerebral vasculature may contribute to IA formation, the specific mechanisms remain to be fully elucidated. Further studies are needed to directly measure bacterial translocation and collagen-binding activity to confirm the role of dental caries in the pathogenesis of IA.

Notably, *S. mutans* ranks among the most recognized oral bacterial species with a well-documented role in hastening tooth decay (26–28). Prior investigations, however, have implicated *S. mutans* (particularly Cnm-positive strains) in the pathophysiology of cerebrovascular events (e.g., intracerebral hemorrhage, aSAH) but not with unruptured IA (12–14, 18). This distinction suggests that while oral pathogens may contribute to the rupture of IA, their role in the formation of unruptured IA remains less clear. Our findings focus specifically on unruptured IA formation. However, it is critical to emphasize that our study did not directly assess *S. mutans* colonization rates, Cnm/Cbm serotypes, or bacterial DNA in saliva samples and IA tissues. Therefore, the mechanistic link between cariogenic bacteria and IA development remains hypothetical and warrants cautious interpretation.

Our study encountered several noteworthy limitations. First, the assessment of caries was conducted using a binary classification system, categorizing teeth as either “present” (decayed, filled, or crowned teeth) or “not present,” instead of employing the decayed-missing-filled teeth index as three distinct variables. Second, it is worth noting that the incidence of IA in our study population was low. Third, our study results exhibited a wide CI, which can be attributed to the relatively small number of IA cases in our dataset. Consequently, we were unable to conduct a comprehensive assessment of the potential dose-response relationship between caries and IA development. Despite our efforts to account for confounding variables in the statistical analyses, it is important to acknowledge the possibility of residual and unmeasured confounding factors.

The association between dental caries and IA suggests that routine oral health assessments (e.g., caries screening, dental radiography) could serve as a low-cost, non-invasive tool to identify individuals at elevated risk for IA. Future studies should directly analyze bacterial DNA (e.g., *S. mutans* Cnm-positive strains) and CBP levels in IA tissues or systemic circulation. This would clarify whether cariogenic bacteria directly contribute to intracranial artery wall remodeling. Besides, the generalizability of our findings may be limited to the Chinese population due to regional differences in oral hygiene practices, dietary habits, and genetic predispositions. Future large-scale, multiethnic studies are needed to validate these associations.

## Conclusion

The presence of surface and/or root dental caries, along with root canal treatment, was associated with IA occurrence. This study is one of the first clinical investigations to assess the correlation between dental caries and IA. Subsequent research endeavors are imperative to corroborate and expand the present findings.

## Data Availability

The raw data supporting the conclusions of this article will be made available by the authors, without undue reservation.
